# Radiotherapy in the treatment of postoperative chylothorax

**DOI:** 10.1186/1749-8090-8-72

**Published:** 2013-04-08

**Authors:** Zsolt Sziklavari, Michael Allgäuer, Georg Hübner, Reiner Neu, Michael Ried, Christian Grosser, Tamas Szöke, Rudolf Schemm, Hans-Stefan Hofmann

**Affiliations:** 1 Department of Thoracic Surgery, Hospital Barmherzige Brüder Regensburg, Prüfeningerstraße 86, 93049, Regensburg, Germany; 2 Department of Radiotherapy, Hospital Barmherzige Brüder Regensburg, Prüfeningerstraße 86, 93049, Regensburg, Germany; 3 Department of Thoracic Surgery, University Regensburg, Franz-Josef-Strauss-Allee 11, 93053, Regensburg, Germany

**Keywords:** Chylothorax, Radiotherapy, Postoperative complication

## Abstract

**Background:**

Chylothorax is characterized by the presence of chyle in the pleural cavity. The healing rate of non-operative treatment varies enormously; the maximum success rate in series is 70%. We investigate the efficacy and outcomes of radiotherapy for postoperative chylothorax.

**Methods:**

Chylothorax was identified based on the quantity and quality of the drainage fluid. Radiation was indicated if the daily chyle flow exceeded 450 ml after complete cessation of oral intake. Radiotherapy consisted of opposed isocentric portals to the mediastinum using 15 MV photon beams from a linear accelerator, a single dose of 1–1.5 Gy, and a maximum of five fractions per week. The radiation target area was the anatomical region between TH3 and TH10 depending on the localization of the resected lobe. The mean doses of the ionizing energy was 8.5 Gy ± 3.5 Gy.

**Results:**

The median start date of the radiation was the fourth day after chylothorax diagnosis. The patients’ mediastinum was radiated an average of six times. Radiotherapy, in combination with dietary restrictions, was successful in all patients. The median time between the end of the radiation and the removal of the chest tube was one day. One patient underwent wound healing by secondary intention. The median time between the end of radiation and discharge was three days, and the overall hospital stay between the chylothorax diagnosis and discharge was 18 days (range: 11–30 days). After a follow-up of six months, no patient experienced chylothorax recurrence.

**Conclusions:**

Our results suggest that radiotherapy in combination with dietary restriction in the treatment of postoperative chylothorax is very safe, rapid and successful. This novel interventional procedure can obviate repeat major thoracic surgery and shorten hospital stays and could be the first choice in the treatment of postthoracotomy chylothorax.

## Background

Chylothorax is characterized by the presence of chyle, which is rich in triglycerides and chylomicrons, in the pleural cavity. There are four typical causes of chylothorax: tumor, trauma, idiopathic and miscellaneous. Chylothorax also complicates up to 3% (0,26 – 3%) of intrathoracic procedures (not including esophagus surgery), especially in patients undergoing radical mediastinal lymph node dissection. Thoracic surgery involving esophageal surgery has a maximum chylothorax incidence of 4% [1]. In cases of chronification, malnutrition and immunologic complications can occur, and a mortality rate of up to 50% has been reported [2]. Currently, the morbidity and mortality associated with chylothorax have improved due to the implementation of more aggressive management strategies.

Conservative treatment of chylothorax includes chest tube drainage and enteral/parenteral dietary management, which is based on the restriction of long chain fatty acids and triglycerides. The daily output from the chest tube is most likely the most important indicator of the patient’s chance of success with conservative treatment. The healing rate of non-operative treatment varies enormously.

If drainage remains >1000 ml/day, surgical intervention has been shown to reverse the adverse effects of chyle [3]. This management strategy decreased the mortality rates for chylothorax [4]. However, especially in cases of postoperative chylothorax, repeated surgical procedures were necessary due to a complicated clinical course. Repeat operations are necessary in up to 72% of cases and include video-assisted thoracic surgery (VATS) and repeat thoracotomy for pleurectomy, talc pleurodesis or ligation of the thoracic duct [3].

Because of the time-consuming conservative treatment and the limited results with interventional and surgical treatment we sought to develop an alternative therapy for postoperative chylothorax. Due to the good outcomes in the treatment of inguinal lymph fistulas with radiotherapy [5] and some well documented case reports describing the successful management of malignant chylothorax with radiotherapy [6,7], we investigated the efficiency and outcome of radiotherapy for postoperative chylothorax.

### Methods

In this retrospective study, we investigated seven patients with postoperative chylothorax who were treated at our department between April, 2010 and October, 2011. Chylothorax was identified based on the quantity and milky aspect of the drainage fluid, and the diagnosis was confirmed based on the documentation of elevated triglyceride levels (>110 mg/dl) in the pleural fluid.

The mean age of the patients (male=4, female=3) was 59 years (range: 27 to 74 years). The cause of chylothorax in all seven cases was thoracotomy. In four of the cases, chylothorax developed following antero-lateral thoracotomy for a lobectomy (n=4), and in 2 cases, chylothorax developed following a wedge resection procedure (n=2); all six of these cases also involved radical lymphadenectomy. One patient underwent a sternotomy for thymectomy without lymph node dissection. A left-sided chylothorax was observed in two patients, and a right-sided chylothorax was observed in five patients. The patient demographics and lung pathologies are summarized in Table 1.

**Table 1:** Patient demographics

	**Patient 1**	**Patient 2**	**Patient 3**	**Patient 4**	**Patient 5**	**Patient 6**	**Patient 7**
Age	74	70	57	59	47	38	62
Sex	Male	Male	Female	Male	Male	Female	Male
Karnofsky-Index <70%	Yes	No	Yes	No	No	No	Yes
Histology	NSCLC Stage IA	NSCLC Stage II A	NSCLC Stage III A	Pseudotumor	Metastasis	Thymoma	Metastasis
Neoadjuvant Therapy	No	No	Yes	No	Yes	No	Yes
Localisation	Right upper lobe	Left lower lobe	Left lower lobe	Left lower lobe	Right midle, lower lobe	Mediastinum	Right upper lobe
Primary Operation	Lobectomy	Lobectomy	Lobectomy	Lobectomy	Wedge- resection	Thymectomy	Wedge- resection
Lympadenectomy	Yes	Yes	Yes	Yes	Yes	No	Yes
Diagnosis, postoperative day	14^th^	6^th^	2^nd^	3^rd^	1^st^	2^nd^	2^nd^
Cholesterol level mg/dl of drainage fluid at Diagnosis°	35	42	45	48	190	65	37
Triglyceride level of drainage fluid mg/dl at Diagnosis°	121	491	371	123	186	1185	224

° Cholesterol level normaly: < 65 mg/dl, Triglyceride level normaly: < 110 mg/dl.

All patients had chest tubes placed after their initial operation. Patients 1, 4 and 7 with highly chyle flow had complete cessation of oral intake (parenteral alimentation), whereas the other four patients were restricted to a fat free diet (exception: medium-chain triglycerides-MCT). Moderate chest tube suction at 20 cm H_2_O was applied. Radiation was indicated if the daily chyle flow exceeded 450 ml after complete oral intake cessation and total parenteral nutrition or fat free diet. A lymphangiography for the identification of the leakage was not performed. The radiotherapy was administered with the written informed consent of the patients. Radiotherapy consisted of opposed isocentric portals to the mediastinum using 15 MV photon beams from a linear accelerator (Synergy, Elekta Stockhom, Sweden), a single dose of 1–1.5 Gy, and a maximum of five fractions per week. The target radiation area was the posterior mediastinum between TH3 and TH10 depending on the localization of the resected lobe. The mean doses of the ionizing energy was 8.5 Gy ± 3.5 Gy. Monitoring of the serum electrolytes, lymphocyte count, albumin, total protein and the patients’ weight was performed.

The medical records were retrospectively reviewed and the following information was collected: patient age, patient sex, clinical course, side of thoracotomy, etiology, radiotherapy course, mean quantity of daily chyle, duration of drainage, length of hospital stay and outcome. All patients were followed up. The mean follow-up period was 6 months (3–16 months). Because of the skewed distributions, we mostly used the median as a location parameter in descriptive statistics. The outcomes are summarized in Table 2.

**Table 2:** Therapy and outcomes

	**Patient 1**	**Patient 2**	**Patient 3**	**Patient 4**	**Patient 5**	**Patient 6**	**Patient 7**
Begin of Radiation after Diagnosis (d)	9^th^	4^th^	4^th^	2^nd^	11^th^	21^st^	3^rd^
Number of Radiations (n)	6	8	8	5	3	3	10
Radiation-Energy (Gy)	6	12	8	5	9	9	10
Max..Flow before Radiation (ml/d)	1100	450	850	800	710	450	1700
Max. Flow after Radiation (ml/d)	0	150	90	90	200	220	0
Day of the chest tube removal after Radiation	1^st^	1^st^	1^st^	2^nd^	8^th^	7^th^	1^st^
Length of hospital after Diagnosis (d)	23	18	15	11	21	30	16
Length of hospital after Radiation (d)	5	3	2	3	13	7	2
Cholesterol after Radiation, mg/dl	35	n.a.	n.a.	67	n.a.	54	50
Triglyceride after Radiation mg/dl	121	n.a.	n.a	40	n.a	126	74
Re-Chylothorax	No	No	No	No	No	No	No
Complications	-	-	-	-	Urosepsis	-	-

## Results

At our institution, postoperative chylothorax occurred in 2.8% of the cases involving thoracic surgery with radical systematic lymphadenectomy. The manifestation of the chylothorax occurred between the 1^st^ and 14^th^ postoperative day (median, 2^nd^ day). The mean pre-radiation daily chyle flow was 800 ml/day (range: 450–1700 ml/day) with a mean concentration of triglycerides of 224 mg/dl (range: 121–1185 mg/dl).

**Figure 1:**
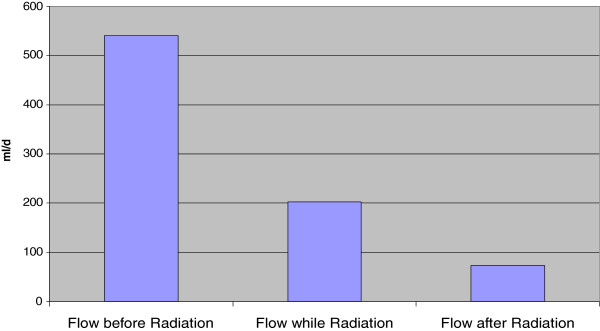
Mean flow of the drainage fluid in ml/die before, while and after radiation.

The median start date of the radiation was on the fourth day (range second-21^st^ day) after diagnosis of the chylothorax. Treatment of the first patient was delayed until the 21^st^ postoperative day because the radio-oncologists were hesitant due to the uncertain outcomes of this novel treatment modality. Radiotherapy, including enteral or parenteral, no-fat diet was successful in all patients. The mediastinum of the patients was radiated a mean of six times (range 3–10 times). During radiotherapy and diet, the mean concentration of triglycerides decreased to 157 mg/dl (range 61–642 ml/day). After radiation, the mean chyle flow was 90 ml/dl (range: 0–150 ml/day) (Figure 1). The median time between the end of the radiation therapy and the removal of the chest tube was one day (range: 1–8 days).

The enteral or parenteral no-fat diet was continued until the end of radiotherapy. One day later, the lymphatic system was activated with a fatty meal. If the drainage fluid remained non-chylous and did not increase, the chest tube was removed.

The majority of the patients had no major complications after radiation. One patient underwent wound healing by secondary intention, and another patient developed urosepsis that was not related to the treatment. The median time between the end of the radiation therapy and discharge was three days (range: 2–13 days), and the overall hospital stay between the diagnosis of chylothorax and discharge was 18 days (range: 11–30 days). After a mean follow-up of six months, no patient experienced chylothorax recurrence.

## Discussion

The present study is the first to show, in a significant number of patients, that postoperative chylothorax can be effectively healed with a combination of radiotherapy and diet.

Chylothorax is a rare condition that results from thoracic duct injury and usually occurs on the right side. The most common causes of chylothorax are malignancy and trauma [8].

Currently, thoracic surgery has replaced physical injury as the leading cause of trauma; esophageal surgery accounts for most of these cases with a maximum incidence of 4% [1]. The incidence of postoperative chylothorax following thoracic surgery without esophageal surgery varies between 0,26% and 3% (Table 3).

**Table 3:** Incidence of secondary chylothorax following thoracic surgery

**Author (year) [Reference]**	**Kind of ymphnodedissection**	**Incidence of postoperative chylothorax (%)**
Bollen et al. 1993 [9]	Mediastinal lymph node dissection	3
Izbicki et al. 1994 [10]	Radical systematic lymphadenectomy	1.2
Cerfolio et al. 1996 [3]	n.a.	0.26
Le Pimpec-Barthes et al. 2002 [11]	Mediastinal lymph node dissection	0.65
Shimizu et al. 2002 [12]	Mediastinal lymph node dissection	2.4
Paul et al. 2009 [13]	Three-field dissection	2.6
Akin et al. 2011 [14]	Mediastinal lymph node dissection	1.9
Own results	Radical systematic lymphadenectomy	2.4

Mediastinal lymphadenectomy seems to be responsible for the higher incidence of postoperative chylothorax after lung cancer resection [9-12,15]. Patients who underwent only sampling of the mediastinal lymph nodes did not develop chylothorax [9,10]. Bollen et al. [9] and Izbicki et al. [10] reported a postoperative chylothorax rate at the beginning of systemic radical mediastinal lymph node dissection of 3% (n=2/65) and 1.2% (n=1/84), respectively. In a larger group of patients (n=1110) undergoing pulmonary resection and systematic mediastinal lymph node dissection for lung cancer, Shimizu et al. reported 27 patients (2.4%) with postoperative chylothorax [12]. Zabeck et al. reported, that in 25 of their 26 postoperative chylothorax cases, complete mediastinal lymph node dissection was performed [15]. In six of our seven cases, complete mediastinal lymph node dissection was performed. At our institution, postoperative chylothorax occurred in 2.8% of the cases involving thoracic surgery with radical systematic lymphadenectomy. The role of pneumonectomy with mediastinal lymph node dissection in the development of chylothorax is unclear. Le Pimpec-Barthes et al. encountered a significantly higher incidence of chylothorax when pulmonary resection consisted of a pneumectomy compared with (bi)lobectomy (5/8 vs. 1/18) [11]. In the patients in the studies conducted by Cerfolio et al. and Shimizu et al., pneumonectomy did not increase the incidence of chylothorax [3,12].

Postoperative chylothorax during the initial operation can be prevented by performing careful dissection and liberal clipping of lymphatic vessels, particularly in the posterior mediastinum. Guo et al. reported that thoracic duct ligation during VATS-esophagectomy for cancer is an effective and safe method for preventing chylothorax [16]. However, there is no consensus or formal guidelines regarding the safe preparation of lymph nodes for lung cancer to prevent postoperative chylothorax.

In patients with postoperative or post-traumatic chylothorax, some authors considered the anatomical location of the thoracic duct injury. Ruan et al. recommends lymphangiography in patients with intractable spontaneous chylothorax [17]. They found that this diagnostic procedure may facilitate the occlusion of the leakage site and the prediction of the occurrence of adverse events due to thoracic duct ligation. We think that radiation therapy for secondary chylothorax does not require any type of leakage detection.

Current treatment modalities for postoperative chylothorax include conservative, interventional and surgical therapies.

The principles of conservative chylothorax treatment include efficient drainage, inflation of the lung to decrease the dead space in the thoracic cavity and to promote adhesion of the lung or pleura to the thoracic duct injury, and cessation of flow (diet/parenteral nutrition) through the thoracic duct to accelerate fistula closure. Conservative treatment must be initiated at the time of diagnosis and results in resolution of the chylothorax in 23% to 70% of cases (Table 4, if n >1).

**Table 4:** Treatment modalities and outcomes for secondary chylothorax

**Treatment**	**Author (year) [Reference]**	**n=**	**Success rate (%)**
***Conservative***			
Diet/parenteral nutrition	Cerfolio et al. 1996 [3]	47	28
Somatostatin-Infusion	Rimensberger et al. 1998 [18]	1	100
Medium Chain Triglycerides Diet	Le Pimpec-Barthes et al. 2002 [11]	26	50
Parenteral nutrition	Shimizu et al. 2002 [12]	26	23
Diet/parenteral nutrition	Chalret du Rieu et al. 2011 [2]	n.a.	70
Diet/parenteral nutrition	Zabeck et al. 2011 [15]	37	32
***Interventional***			
Percutaneous Embolization	Cope 2004 [19]	60	65
Pedal Lymphangiography	Le Pimpec-Barthes et al. 2002 [11]	7	57
Pleurodesis (OK-432)	Shimizu et al. 2002 [12]	15	87
Pleurodesis (talc)	Paul et al. 2009 [13]	6	83
Pleurodesis (talc)	Akin et al. 2011 [14]	26	73
Direct lymphangiography	Schoellnast et al. 2011 [20]	2	50
***Surgery***			
Duct ligation	Cerfolio et al.1996 [3]	32	94
Duct ligation	Shimizu et al. 2002 [12]	5	100
Duct ligation/Loc. Suture	Le Pimpec-Barthes et al. 2002 [11]	5	83
Duct ligation	Paul et al. 2009 [13]	22	95
Duct ligation	Akin et al. 2011 [14]	7	100

Interventional/surgical management should be adopted if conservative management fails. The interventional therapies include VATS for pleurodesis, percutaneous lymphangiography and radiotherapy. Schoellnast et al. reported a 50% success rate after needle disruption of the cisterna chyli with subsequent resolution of the chylothorax [20]. Cope found that two thirds of patients presenting with life-threatening chylothorax can be safely treated with percutaneous transabdominal thoracic duct blockage [19]. Promotion of adhesions of the lung and pleura to the thoracic duct injury by pleurodesis may play an important role in the treatment of chylothorax. Talc pleurodesis is successful in 73% to 83% of all cases [13,14]. The injection of OK-432, which is a heat- and penicillin-treated lyophilized preparation of the Su strain of Streptococcus pyogenes, into the thoracic cavity is effective and safe in managing postoperative chylothorax and exhibited a success rate of 87% [12]. Other therapeutic options such as the intrapleural instillation of octreotide/somatostatin, streptokinase or fibrin glue therapy were only published as case reports [18,21,22].

Surgical treatment generally involves ligation of the thoracic duct. Thoracic surgeons often recommend reoperation to ligate the thoracic duct when conservative therapy fails [3,12,15]. Surgical intervention offers better results than conservative management when the daily chyle flow exceeds 500 ml/day in adults [12]. Zabeck et al. found that in postoperative chylothorax with a high flow of >900 ml/day, repeat surgery should be performed as soon as possible because conservative management is likely to be unsuccessful [15].

Radiotherapy of inguinal lymph fistulas is a well-established, effective and well-tolerated treatment option. Because fistulas completely close after radiotherapy, this procedure is a good alternative to other conservative treatment modalities [5]. The motivations for our study were some well documented case reports that described the successful management of malignant chylothorax with radiotherapy [6,7].

Analogously, there are several potential advantages of using radiation to treat chylothorax. The early use of radiation therapy may rapidly arrest the progression of chylothorax and allow the patient to avoid invasive procedures. Up to 11% of the patients with postoperative chylothorax underwent repeat surgical procedures due to complications, and 9% experienced recurrence of their chylothorax [15]. Shimizu et al. reported that complications following VATS pleurodesis and repeat thoracotomy occurred in 15% of cases [12].

Accordingly, the median length of hospital stay was shorter in our group (median, 18 [range 11–30] days) compared with the group that underwent (redo) operation in the studies of Kashiowanoha (19 [range 12–36] days) and Heidelberg (26 [range 15–205] days), respectively [12,15].

The major disadvantage to using radiation therapy for chylothorax is the possibility for acute and long-term side effects that vary by treatment site. On the other hand, no patient developed in our study radiation side effect (e.g. pneumonitis) after the low-dose radiotherapy. While moderate/severe radiation-induced side effects are rare, the potential for secondary malignancy and growth restriction should be considered before administering high-dose radiation therapy especially to children.

The great advantages of radiation therapy with a maximum of 12 Gy include the low incidence of acute and long-term side effects. After a mean follow-up of 6 months, no recurrent chylothorax or other complications were observed in any of the seven patients. All of these patients reported a very good quality of life in an outpatient interview.

Future studies should investigate the required minimum energy that is required to eliminate the chyle leakage.

### Study limitation

The limitation of our study was the small number of patients.

## Conclusion

**Figure 2:**
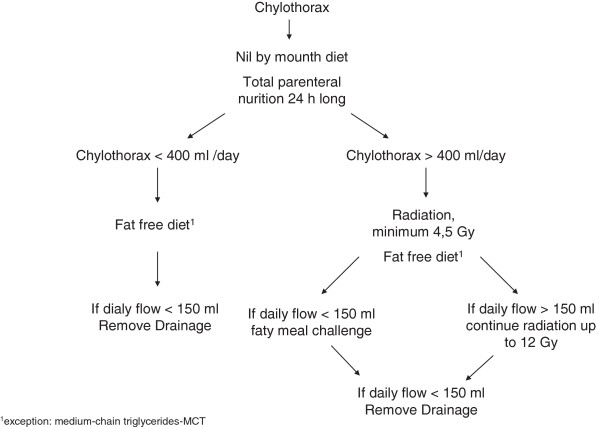
Therapy recommendation for postoperative chylothorax.

Our results suggest that radiotherapy in combination with diet in the treatment of postoperative chylothorax is very safe, rapid and successful. Our therapy recommendation is summarized in Figure 2. This interventional procedure can obviate repeat major thoracic surgery and shorten hospital stays and could be the first choice in the therapy of postthoracotomy chylothorax.

## Competing interest

All co-authors have seen and agreed with the contents of the manuscript and there is no financial interest to report.

## Authors' contributions

CG, MA, and GH participated in the design of the study. TS and RS participated in the sequence alignment and drafted the manuscript. ZS, HH and MR conceived of the study and participated in its design and coordination. RN participated in surgery. All authors read and approved the final manuscript.

## References

[B1] McGrath EE Blades Z Anderson PB Chylothorax: aetiology, diagnosis and therapeutic options Respir Med 2010 104 1 8 10.1016/j.rmed.2009.08.010 19766473

[B2] Chalret Du Rieu M Mabrut JY Management of postoperative chylothorax J Visc Surg 2011 148 5 e346 e352 10.1016/j.jviscsurg.2011.09.006 22033151

[B3] Cerfolio RJ Allen MS Deschamps C Trastek VF Pairolero PC Postoperative chylothorax J Thorac Cardiovasc Surg 1996 112 1361 1365 10.1016/S0022-5223(96)70152-6 8911335

[B4] Khurana H Mishra S Jain R Goyal GN Bhatnagar S Management of post-operative chylothorax in a patient with carcinoma of thyroid and lymphadenopathy - a case report Middle East J Anesthesiol 2009 20 121 123 19266840

[B5] Dietl B Pfister K Aufschläger C Kasprzak PM Radiotherapy of inguinal lym-phorrhea after vascular surgery. A retrospective analysis Strahlenther Onkol 2005 181 396 400 10.1007/s00066-005-1364-0 15925983

[B6] Gerstein J Kofahl-Krause D Fruhauf J Bremer M Complete remission of a lymphoma-associated chylothorax by radiotherapy of the celiac trunk and thoracic duct Strahlenther Onkol 2008 184 484 487 10.1007/s00066-008-1840-4 19016028

[B7] Cigarral C Montero A Salas C Rodriguez G de la Torre A Chylothorax due to metastasic prostate carcinome: an unusual complication Clin Transl Oncol 2009 11 11 767 769 10.1007/s12094-009-0441-8 19917542

[B8] Doerr CH Allen MS Nichols FC 3rd Ryu JH Etiology of chylothorax in 203 patients Mayo Clin Proc 2005 80 867 870 10.4065/80.7.867 16007891

[B9] Bollen EC Van Duin CJ Theunissen PH Vt Hof-Grootenboer BE Blijham GH Mediastinal lymph node dissection in resected lung cancer: morbidity and accuracy of staging Ann Thorac Surg 1993 v 961 966 8385446 10.1016/0003-4975(93)90126-3

[B10] Izbicki JR Thetter O Habekost M Karg O Passlick B Kubuschok B Busch C Haeussinger K Knoefel WT Pantel K Radical systematic mediastinal lymphadenectomy in non-small cell lung cancer: a randomized controlled trial Br J Surg 1994 81 229 235 10.1002/bjs.1800810223 8156344

[B11] Le Pimpec-Barthes F D`Attellis N Dujon A Legman P Riquet M Chylothorax complicating pulmonary resection Ann Thoracic Surg 2002 73 1714 1719 10.1016/S0003-4975(02)03570-1 12078758

[B12] Shimizu K Yoshida J Nishimura M Takamochi K Nakahara R Nagai K Treatment strategy for chylothorax after pulmonary resection and lymph node dissection for lung cancer J Thorac Cardiovasc Surg 2002 124 499 502 10.1067/mtc.2002.124386 12202866

[B13] Paul S Altorki NK Port JL Stiles BM Lee PC Surgical management of chylo-thorax Thorac Cardiovasc Surg 2009 57 226 228 10.1055/s-0029-1185457 19670117

[B14] Akin H Olcmen A Isgorucu O Denizkiran I Dincer I Approach to patients with chylothorax complicating pulmonary resection Thorac Cardiovasc Surg 2012 60 2 135 139 21557161 10.1055/s-0030-1270990

[B15] Zabeck H Muley T Dienemann H Hoffmann H Management of chylothorax in adults: when is surgery indicated? Thorac Cardiovasc Surg 2011 59 243 246 10.1055/s-0030-1250374 21425049

[B16] Guo W Zhao YP Jiang YG Niu HJ Liu XH Ma Z Wang RW Prevention of postoperative chylothorax with thoracic duct ligation during video-assisted thoracoscopic esophagectomy for cancer Surg Endosc 2012 26 5 1332 1336 10.1007/s00464-011-2032-3 22044984

[B17] Ruan Z Zhou Y Wang S Zhang J Xu W Clinical use of lymphangiography for intractable spontaneous chylothorax Thorac Cardiov Surg 2011 59 7 430 435 10.1055/s-0030-1271031 21557160

[B18] Rimensberger PC Müller-Schenker B Kalangos A Beghetti M Treatment of a persistent postoperative chylothorax with somatostatin Ann Thorac Surg 1998 66 253 254 10.1016/S0003-4975(98)00361-0 9692478

[B19] Cope C Management of chylothorax via percutaneous embolization Curr Opin Pulm Med 2004 10 4 311 314 10.1097/01.mcp.0000127903.45446.6d 15220758

[B20] Schoellnast H Maybody M Getrajdman GI Bains MS Finley DJ Solomon SB Computed tomography-guided access to the cisterna chyli: introduction of a technique for direct lymphangiography to evaluate and treat chylothorax Cardiovasc Intervent Radiol 2011 34 Suppl 2 S240 S244 20396888 10.1007/s00270-010-9851-9

[B21] Kuan YC How S Ng T Fauzi A Intrapleural streptokinase for the treatment of chylothorax Respir Care 2011 56 1953 1955 10.4187/respcare.01207 21682984

[B22] Akaogi E Mitsui K Sohara Y Endo S Ishikawa S Hori M Treatment of postoperative chylothorax with intrapleural fibrin glue Ann Thorac Surg 1989 48 116 118 10.1016/0003-4975(89)90193-8 2475070

